# Femoral bone structure and mechanics at the edge and core of an expanding population of the invasive frog *Xenopus laevis*

**DOI:** 10.1242/jeb.246419

**Published:** 2024-07-11

**Authors:** Maïtena Dumont, Anthony Herrel, Julien Courant, Pablo Padilla, Ron Shahar, Joshua Milgram

**Affiliations:** ^1^ Laboratory of Bone Biomechanics, Koret School of Veterinary Medicine, The Robert H. Smith Faculty of Agriculture, Food and Environment, PO Box 12, 7610001 Rehovot, Israel; ^2^ Max-Planck Institute for Sustainable Materials, MPISM, D-40237, Düsseldorf, Germany; ^3^ UMR 7179 CNRS/MNHN, Département Adaptations du Vivant, Bâtiment d'Anatomie Comparée, 55 rue Buffon, 75005 Paris, France; ^4^ Department of Biology, Evolutionary Morphology of Vertebrates, Ghent University, Ghent 9000, Belgium; ^5^ Department of Biology, University of Antwerp, Wilrijk 2610, Belgium; ^6^ Naturhistorisches Museum Bern, 3005 Bern, Switzerland; ^7^ Laboratory of Ecology and Conservation of Amphibians (LECA), Freshwater and Oceanic Science Unit of Research (FOCUS), University of Liège, Liège, Liege 4000, Belgium

**Keywords:** *Xenopus laevis*, Femora, Bone cortical analysis, Biomechanical properties, Invasive species

## Abstract

Understanding how living tissues respond to changes in their mechanical environment is a key question in evolutionary biology. Invasive species provide an ideal model for this as they are often transplanted between environments that differ drastically in their ecological and environmental context. Spatial sorting, the name given to the phenomenon driving differences between individuals at the core and edge of an expanding range, has been demonstrated to impact the morphology and physiology of *Xenopus laevis* from the invasive French population. Here, we combined a structural analysis using micro-CT scanning and a functional analysis by testing the mechanical properties of the femur to test whether the increased dispersal at the range edge drives differences in bone morphology and function. Our results show significant differences in the inner structure of the femur as well as bone material properties, with frogs from the centre of the range having more robust and resistant bones. This is suggestive of an energy allocation trade-off between locomotion and investment in bone formation, or alternatively, may point to selection for fast locomotion at the range edge. Overall, our results provide insights on the growth of the long bones and the formation of trabecular bone in frogs.

## Teaser

**Highlighted Article:** Differences of inner structure and bone material properties of frog femora are due to spatial sorting.

## INTRODUCTION

Invasive species are among the principal causes of the current loss of biodiversity worldwide (Mollot et al., 2017; Simberloff et al., 2013). However, invasive species also inadvertently provide an exceptional model to study and understand the evolutionary mechanisms underlying range expansion (Burton et al., 2010; Tingley et al., 2014) and the mechanisms driving the evolution of dispersal phenotypes (Philips et al., 2006; Shine et al., 2011). The African clawed frog, *Xenopus laevis*, a predominantly aquatic frog from sub-Saharan Africa, has become invasive on a global scale (Tinsley et al., 2009; Measey et al., 2012) and was introduced into France in the early 1980s (Fouquet, 2001). Since its introduction, the species has spread rapidly, and is now present in at least five provinces in France (Vimercati et al., 2020). Although primarily aquatic, animals also disperse overland during heavy rainfall (Courant et al., 2019a).

Previous studies have demonstrated significant differences in the morphology and physiology in frogs from the core and edge of the range in the French population; a finding attributed to spatial sorting (Courant et al., 2017, 2019a; Louppe et al., 2017; Padilla et al., 2019, 2020). Spatial sorting is a consequence of rapid population expansion, resulting in individuals with a different phenotype being present at the edge of the range. This subsequently results in non-random mating with other individuals that exhibit a similar dispersal phenotype (i.e. only animals that are good dispersers will encounter each other at the edge of the invasive range and will thus mate; see Burton et al., 2010; Shine et al., 2011; Chuang and Peterson, 2016). Individuals at the edge of the range of the invasive population of *X. laevis* in France show a higher *in vivo* endurance capacity (Louppe et al., 2017), a lower standard metabolic rate (SMR; Louppe et al., 2018), and a lower investment in reproduction (Courant et al., 2017) when compared with individuals from the core of the range. In addition, *X. laevis* from the edge of the range have longer legs and stronger hind limb muscles with a greater physiological cross-sectional area (Louppe et al., 2017; Padilla et al., 2019). These differences at least partly account for the greater dispersal rates and dispersal distances observed for individuals at the range edge (Courant et al., 2019a).

In addition, sexual dimorphism has been observed in *X. laevis* (Louppe et al., 2017; Padilla et al., 2019). For example, despite their smaller size compared with females, male *X. laevis* have relatively larger and more powerful muscles (Padilla et al., 2019), relatively longer legs and greater stamina than females (Louppe et al., 2017). These differences have been suggested to be responsible for the greater dispersal observed in males compared with females (Courant et al., 2019a).

Bone is a living tissue that responds to the external forces exerted upon it by modeling (e.g. Currey, 2002; Kular et al., 2012; Stoltz et al., 2018). Given the known sexual dimorphism, and differences in morphology, physiology and endurance in individuals from the edge and core of the range, we predicted differences in bone structure and mechanical properties between these individuals. Specifically, we predicted that individuals from the edge would show long bone adaptations consistent with the increased frequency and higher loads to which they are subjected during locomotion, and that these differences would be more pronounced in males. Alternatively, the energy allocated to locomotion and muscle development may impact the energy available to bone growth, resulting in the opposite pattern. To test these hypotheses, we focused on the femur, as the extensor muscles attached to it are the primary source of the power needed during jumping (Prikryl et al., 2009; Padilla et al., 2019). We tested for differences in cortical thickness, mean cross-sectional area of the bone, its second moment of area, bone mineral density, Young's modulus, and the bending yield stress and strain between individuals from the core and the edge of the range, as well as between males and females.

## MATERIALS AND METHODS

*Xenopus laevis* Daudin 1802 used in this study were harvested from individuals collected by Courant et al. (2019a). Animals were collected and euthanized under permit from the Préfet des Deux-Sèvres. Euthanasia was performed by injection of a lethal dose of sodium pentobarbital. The study sites were located in the expanding range of *X. laevis* in Western France (Courant et al., 2019a). The edge and core sites were defined as in Courant et al. (2019a) and Padilla et al. (2019). Frogs were captured in 18 ponds from May to October 2014 and during spring 2016. The left femora were dissected free of soft tissues and stored in ethanol (70% solution). In total, 36 samples were investigated (8 females and 10 males collected from core sites; 9 females and 9 males collected from the edge sites). The age of the individuals ranged from <1 year to 6 years (Courant et al., 2019b). Despite differences in core and edge populations (e.g. in endurance capacity, metabolic rate and muscle cross-section area), no differences in growth rate were present (Courant et al., 2019b). Consequently, we used femur length here as a proxy for the age and size of the animals.

### Micro-CT scanning

All bones were scanned using a desktop Micro-CT scanner (Skyscan® 1174, micro-CT scanner, Skyscan, Belgium). Each bone was placed in a plastic tube and stabilized in agar. The X-ray source was set at 50 kVp and 800 μA. All specimens were scanned over 180 deg with a rotation step of 0.4 degrees and exposure time of 4 s. A 0.25 mm aluminium filter was used to decrease beam hardening effects. The entire femur was scanned at an isotropic pixel spacing of 33.6 μm. The diaphysis of one of the samples was scanned at higher resolution (9.6 μm) and compared with the same femur scanned at lower resolution to ensure the quality and reliability of the cortical diaphysis measurements of the scan at lower resolution. Epiphyses were scanned at an isotropic pixel spacing between 7.5 and 12.2 μm (depending on the size of the femur).

Scans were reconstructed and analysed using commercial software (NRecon^®^ v. 1.6.9.18, Bruker^®^, Kontich, Belgium and CT analyzer^®^, Bruker^®^, Kontich, Belgium, respectively). A region of interest (ROI) of 150 slices, centered on the mid-diaphysis, was used for the cortical analysis. The mid-diaphysis is chosen by convention and according to previous research showing that this is the location where peak strains are highest (e.g. Biewener and Taylor, 1986). The trabecular analysis was carried out on the distal and proximal epiphyses. ROIs of 250 slices starting at the wedge-shaped margin of the periosteal bone (as described by Rozenblut and Ogielska, 2005) were used. Two phantoms with known density (0.25 g cm^−3^ and 0.75 g cm^−3^) were scanned using the identical parameters used for scanning the femora allowing the calculation of the bone mineral density (BMD) of the cortical diaphysis. Morphometric analysis and BMD evaluation was carried out using dedicated software (Skyscan^®^ CT Analyser, version 1.15.4, Skyscan, Belgium). Three dimensional representations of the various bones were obtained using Amira (FEI, version 5.6.0, Hillsboro, Oregon, USA).

### Bone characterization

The results of the cortical analysis for all femora are listed in Table 1, using measurements and terminology similar to that of Bouxsein et al. (2010). The results of the cortical analysis included the total cross-sectional tissue area (CSA_tissue_, in mm^2^), the cortical bone cross-sectional area (CSA_bone_, in mm^2^), the average cortical thickness (*L*_cortex_, in mm), the maximum and minimum average second moment of area (*I*_min_ and *I*_max_, in mm^4^), the mean polar moment of area (*I*_polar_, in mm^4^) and the mean eccentricity (*e*). For the trabecular analyses only a subset of individuals was used (see Tables S1 and S4) and only the distal epiphysis was used as proximal epiphyses often had few trabeculae. The variables extracted for the trabecular analysis included bone volume (*V*_bone_, in mm^3^), tissue volume (*V*_tissue_, in mm^3^) bone volume fraction (*V*_bone_/*V*_tissue_, in %), trabecular number (*N*_Tb_, in mm^−1^), trabecular thickness (*L*_Tb_, in mm), trabecular separation (*L*_Tb separation_, in mm).

**Table 1.**
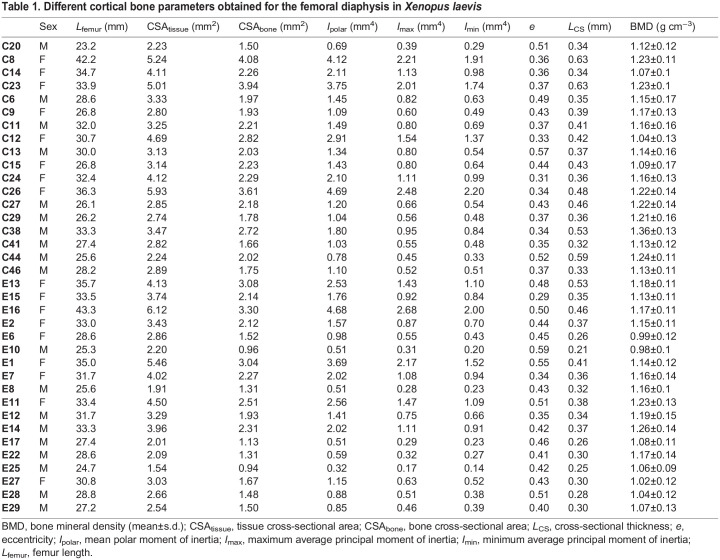
Different cortical bone parameters obtained for the femoral diaphysis in *Xenopus laevis*

### Three-point bending

Three-bending mechanical testing was conducted not only for comparative purposes – the largest and most comprehensive dataset on mechanical properties of long bones was obtained by this method (Erickson et al., 2002) – but bending is also the primary loading direction and source of bone strain in limb bones of tetrapods (e.g. Biewener and Taylor, 1986). Moreover, Currey demonstrated that bones tend to fail on the tensile side during bending (Currey, 2002).

Three-point bending was performed on six femora from each group using a custom-built micro-mechanical testing device. The caudal aspect of the diaphysis of each bone was positioned on the supports of the stationary anvil such that the support of the moving anvil contacted the bone at its midpoint. The distance between the stationary supports was 15 mm (note that we did not adjust the span based on femur length which may have induced some bias in our results). A preload of 1.5–1.8 N was applied to the sample prior to testing. Each specimen was tested under displacement control and at a rate of 300 μm min^−1^ to fracture, which was characterized by a sudden decrease in load. The loading rate was lower than in previous studies as these are predominantly aquatic frogs for which loading rates can be assumed to be lower. Yet, this remains hypothetical as no *in vivo* loading rates have been measured or estimated for this species. The force–displacement data obtained were then converted to stress (σ) and strain (ε) using beam theory where:
(1)

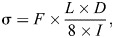
where *F* is the load applied (N), *L* the span used in the bending test (mm), *D* the diameter at the midpoint of the femur (mm) and *I* the inner second moment of area (mm^4^). The diameter at the midpoint of the femur (*D*) was the average cortical diameter determined from 10 different CT images:
(2)

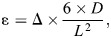
where Δ is the displacement (mm), *D* the diameter of the bone (mm) and *L* the span of the stationary anvil (mm).

The Young's modulus (N mm^−2^ or MPa) of the bone was obtained from the slope of the linear part of the stress–strain curve. In addition, the stress and strain at the yield point and the point of fracture were also determined. The yield point was determined as the point which marked the transition between the linear part and the plastic area of the stress–strain curve (e.g. Turner, 2006; Sharir et al., 2008): it was determined by successive steps on the curve by obtaining linear regression. At this yield point, the yield stress (σ_y_) and yield strain (ε_y_) are determined. Fracture stress and strain represent the point where the bone breaks. The bones were stored in ethanol since the samples were harvested in 2004 and 2006. Whereas prior work suggested that mechanical properties of bones stored in ethanol for less than 14 days and up to 100 days (e.g. respectively Turner and Burr, 1993; Linde and Sorensen, 1993) are not affected, this does not seem the case for long storage as in our case. Vesper et al. (2017), did indeed notice an increase in bone stiffness and elastic modulus after long storage (∼7 weeks) in ethanol. Consequently, we may overestimate stiffness and elastic modulus. However, since all bones were subjected to the same condition, results should be consistent and comparable between different groups (core versus edge).

### Statistical analysis

All data were log_10_-transformed before analyses to ensure normality and homoscedasticity of the data using Shapiro–Wilk's and Levene's tests (see Tables S2 and S3). All variables were normally distributed and all showed homogeneity of variances except yield stress (Table S3).

First, we ran a univariate analysis of covariance (ANCOVA) with femur length as a covariate to test for differences in bone mineral density between populations and sexes. We also tested interaction between population and sex. Next, we ran multivariate analyses of covariance (MANCOVA) with femur length as a covariate on three different data sets: (1) cortical bone parameters, (2) trabecular measures of the distal epiphysis, and (3) the biomechanical measurements (Young's modulus, stress at/to fracture, yield stress and yield strain). Subsequently, we ran univariate ANCOVAs with sequential Bonferroni correction to test which variables differed between populations or sexes. All models included interaction terms. All tests were two-tailed as we had no *a priori* predictions concerning the directionality of possible differences. Means±s.d. are reported.

## RESULTS

### Cortical diaphysis analysis

**Fig. 1.**
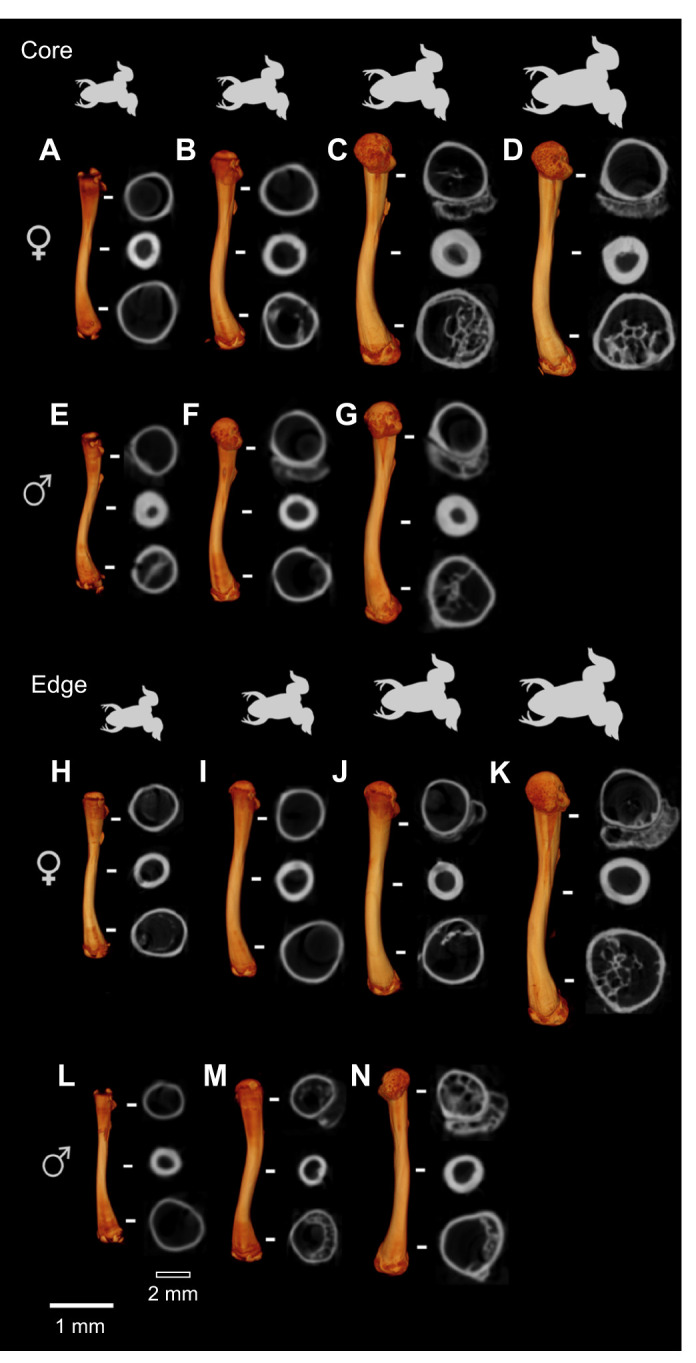
**Reconstructions of selected representative femurs from individual *Xenopus laevis* of increasing size.** (A–D) female core; (E–G) male core; (H–K) female edge; (L–N) male edge). Corresponding cross sections show details of cortical bone at three locations in each bone indicated by the white horizontal stripes. Female core: (A) C15 (femora length, 25 mm), (B) C24 (32.42 mm), (C) C26 (36.34 mm), (D) C8 (42.22 mm); male core: (E) C44 (25.58 mm), (F) C29 (26.24 mm), (G) C38 (33.3 mm); female edge: (H) E6 (28.55 mm), (I) E7 (31.7 mm), (J) E15 (33.47 mm), (K) E16 (43.27 mm); male edge: (L) E25 (24.69 mm), (M) E28 (28.8 mm), (N) E14 (33.33 mm). Scale bars: 1 mm (white), corresponding to the images of the bones; 2 mm (black), corresponding to the bone cross-sections.

**Fig. 2.**
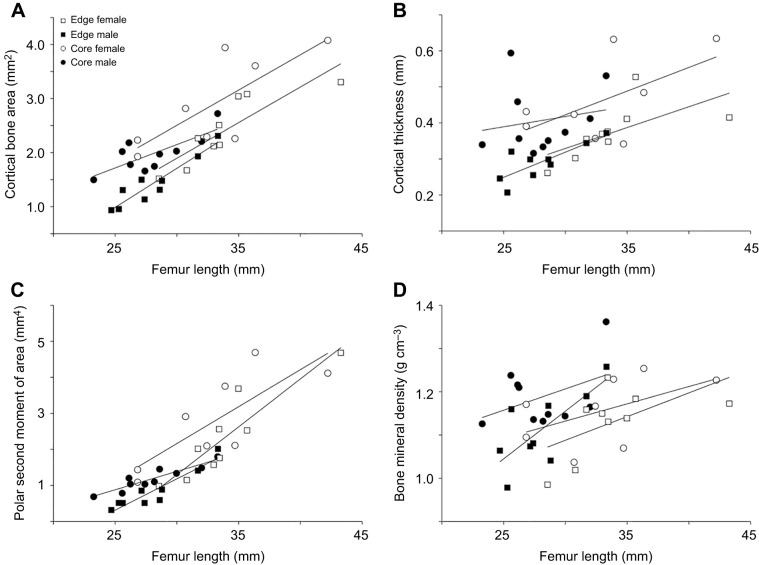
**Comparison of cortical bone parameters as a function of femoral length.** (A) Cross-sectional bone area (CSA_bone_; mm^2^), (B) Cortical thickness (*L*_cortex_; mm), (C) Mean polar second moment of area (*I*_polar_; mm^4^), (D) bone mineral density (BMD; g cm^−3^) between the edge (square) and core (circle) populations for males (black) and females (white). Trend lines have been added to the graphs.

Figs 1 and 2 illustrate representative long bones of male and female frogs from the core and the edge populations. Differences in the cortical diaphysis are not obvious. The cortical thickness increased with bone length in male and female frogs and differences appeared when comparing individuals from the core compared with those from the edge of the range (Figs 1 and 2).

Femur length had a significant impact on the cortical diaphysis (Wilks' lambda=0.23; *F*_8,24_=10.29; *P*<0.001). The analysis also showed significant population effects (Wilks' lambda=0.40; *F*_8,24_=4.45; *P*=0.002). The sex effect (Wilks' lambda=0.59; *F*_8,24_=2.05; *P*=0.08) was marginally non-significant and the interaction between sex and population (Wilks' lambda=0.78; *F*_8,24_=0.89; *P*=0.56) was not significant. Subsequent ANCOVAs showed differences between males and females (Tables 2 and 3) with females typically having larger values than males (e.g. mean total cross-sectional tissue area, mean cross-sectional bone area, mean polar second moment of area). Similarly, differences between populations were significant for all variables except the mean eccentricity (Tables 2 and 3) with individuals from the core of the range showing higher values compared with those of the edge of the range.

**Table 2.**
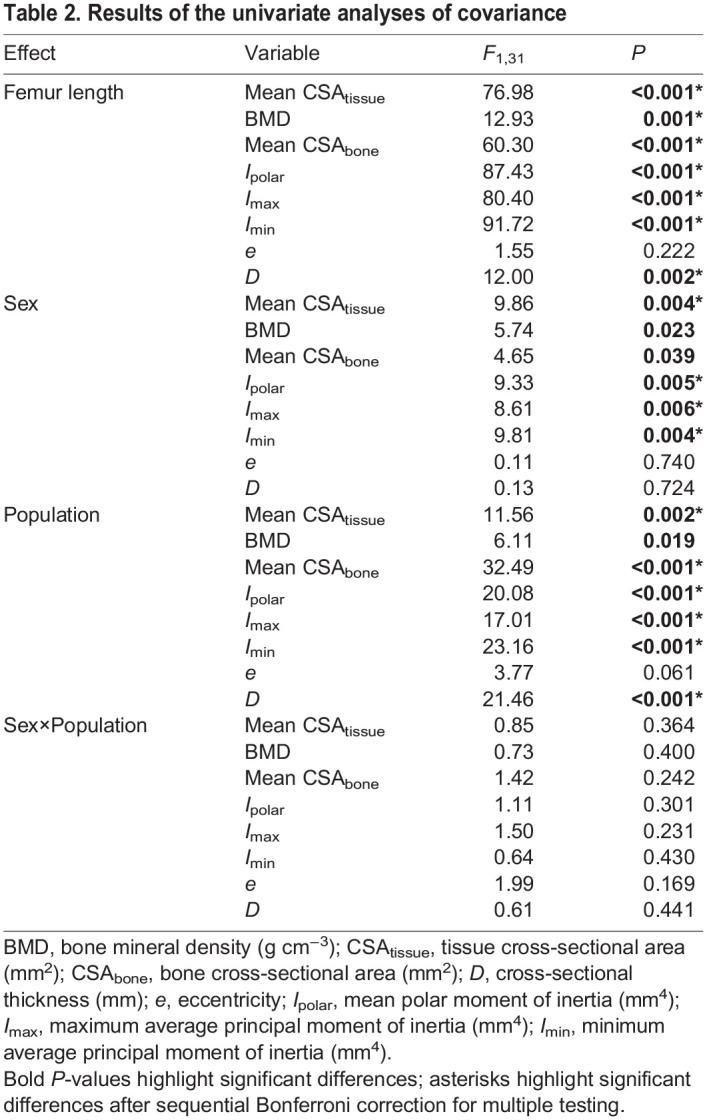
Results of the univariate analyses of covariance

**Table 3.**
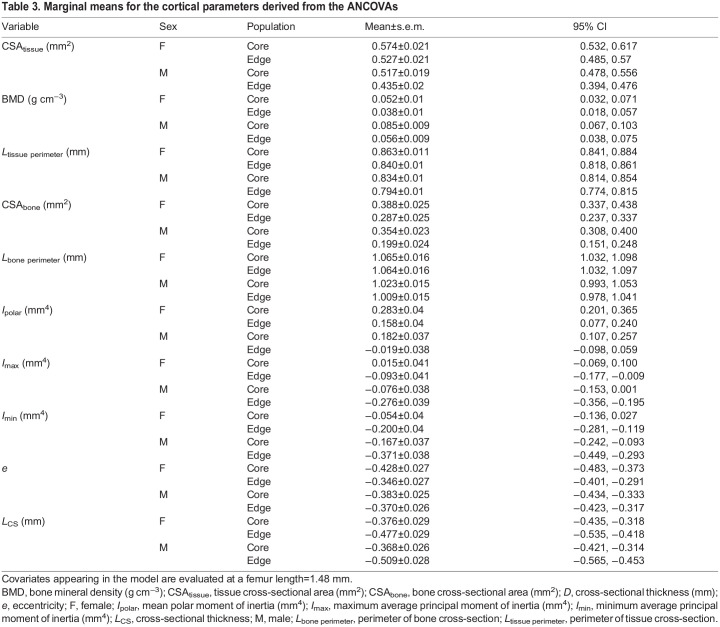
Marginal means for the cortical parameters derived from the ANCOVAs

### Bone mineral density

The average BMD for all femora was 1.15±0.08 g cm^–3^ (Fig. 2D, Table 1) with the lowest values being below 1 g cm^–3^ for two edge samples. There was a significant effect of femur length on bone mineral density (*F*_1,31_=12.93; *P*=0.001; Fig. 2D) with larger bones being more mineralized. The analysis of covariance detected significant sex differences (*F*_1,31_=5.74; *P*=0.023) and differences between populations (*F*_1,31_=6.12; *P*=0.0190, but the interaction between sex and population was not significant (*F*_1,31_=0.73; *P*=0.40). Inspection of the marginal means showed that females had a significantly lower BMD than males and that individuals from the edge of the range had lower BMD than those of the core for a given femur length.

### Trabecular epiphyses bone analysis

**Fig. 3.**
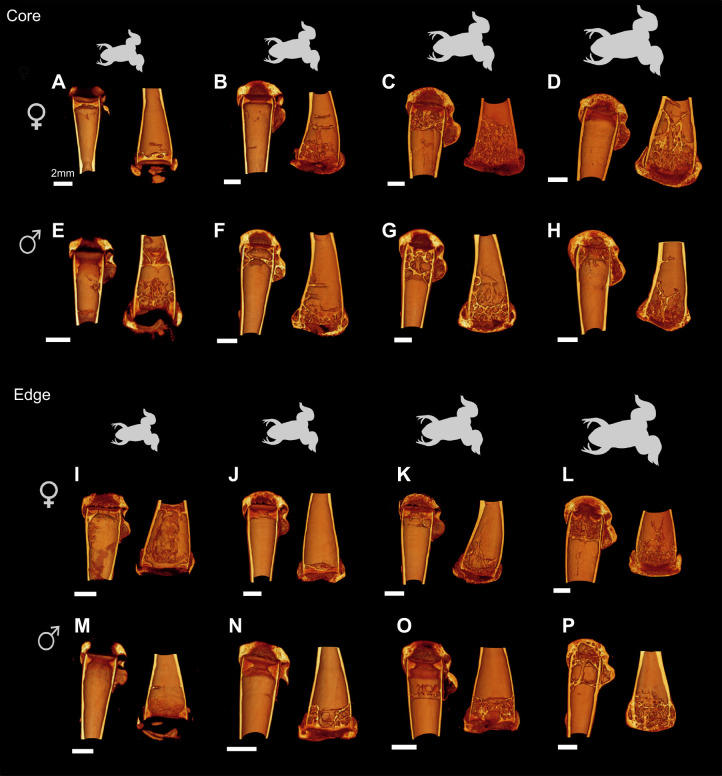
**Illustration of the trabecular bone present in a longitudinal cut of *X. laevis* femora epiphyses.** The proximal (left) and distal (right) epiphyses of selected femora for the core population (A–H) and edge (I–P) for each sex, ranked by increasing femur length (from left to right). Female core: (A) C15 (femur length, 26.82 mm), (B) C24 (32.42 mm), (C) C23 (33.9 mm), (D) C8 (42.22 mm); male core: (E) C44 (25.58 mm), (F) C29 (26.24 mm), (G) C11 (32 mm), (H) C38 (33.3 mm). Female edge: (I) E6 (28.55 mm), (J) E7 (31.7 mm), (K) E15 (33.47 mm), (L) E16 (43.27 mm); male edge: (M) E25 (24.69 mm), (N) E17 (27.39 mm), (O) E28 (28.8 mm), (P) E14 (33.33 mm).

**Fig. 4.**
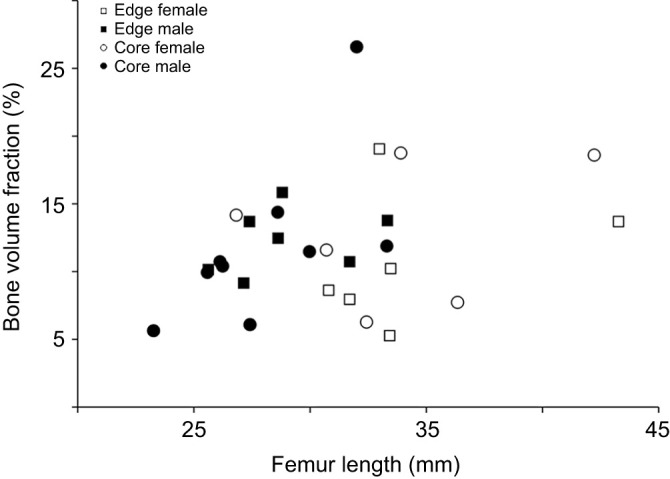
**Scatterplot of bone volume relative to total tissue volume against femur length for the distal epiphyses of *X. laevis* femora.** Bone volume fraction (*V*_bone_/*V*_tissue_, in %) in edge individuals is represented by squares and core individuals by circles. Males are indicated in black and females in white. Raw data can be found in Table S6.

Longitudinal sections of the epiphyses of male and female frogs from the core and edge populations are illustrated in Fig. 3. It can be clearly seen that some femora did not show any trabecular bone in either the proximal or the distal epiphyses (Tables S1 and S4). The trabecular bone parameters analyzed are presented in Fig. 4 and Table S4. The bone volume fraction for the proximal and distal epiphysis ranged from 5% to 25% (Fig. 4; Fig. S1). The trabecular thickness in the proximal epiphysis varied between 100 and 150 µm, and between 100 µm and 160 µm in the distal epiphysis (Fig. S2). As the trabecular bone observed was mostly present in the distal epiphysis of older individuals (Fig. 3; Table S1), our statistical analysis was restricted to this region.

Femur length significantly impacted the distal trabecular architecture (Wilks' lambda=0.54; *F*_4,20_=4.29; *P*=0.011). Effects were significant for trabecular volume (*F*_1,23_=13.31; *P*=0.001), bone volume (*F*_1,23_=16.76; *P*<0.001), the ratio of bone volume to trabecular volume (*F*_1,23_=4.76; *P*=0.040) and trabeculae number (*F*_1,23_=4.47; *P*=0.046). However, neither sex (Wilks' lambda=0.86; *F*_4,20_=0.82; *P*=0.53) nor population (Wilks' lambda=0.72; *F*_4,20_=1.95; *P*=0.14), nor their interaction (Wilks' lambda=0.87; *F*_4,20_=0.76; *P*=0.56) impacted the trabecular bone structure. Consequently, neither populations nor sexes differed in the trabecular structure of the distal epiphysis (see also Fig. S1).

### Three-point bending analysis

**Fig. 5.**
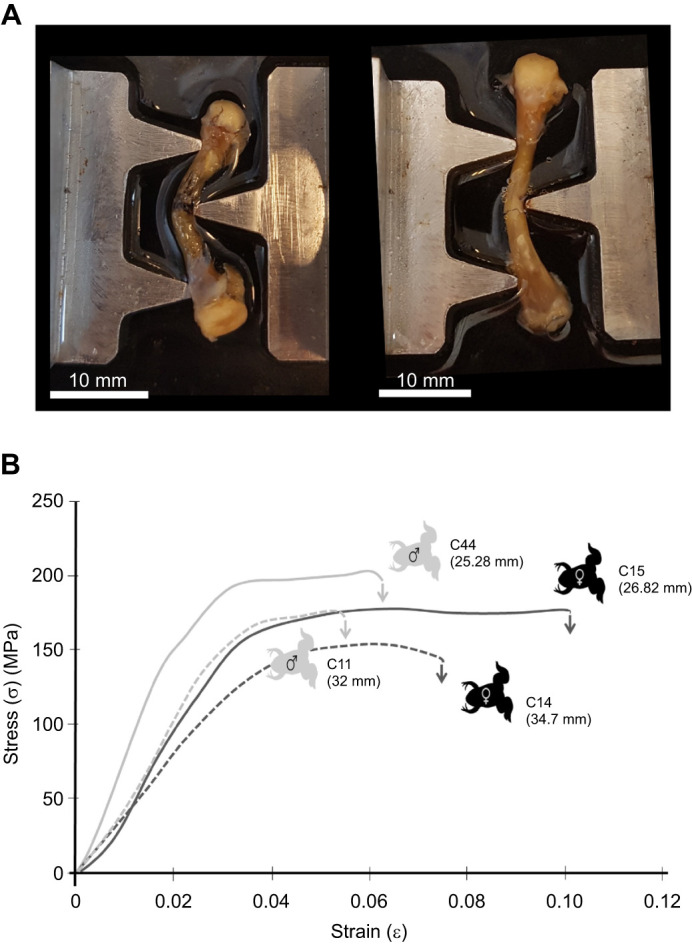
**Measurement of strain and stress of *X. laevis* femora in a three-point bending test.** (A) Three-point bending test of a femur harvested from a male (left: individual C44; femur length of 25.6 mm) and female (right: individual C26; femur length of 36.34 mm). (B) Graph of the strain–stress curves for four representative femora: males are indicated in grey, females in black. The dashed lines represent larger individuals and the solid lines smaller ones. Arrows indicate fracture of the sample. Femur lengths are indicated below the individual number. C, core; E, edge. Raw data can be found in Table 3.

The results from the bending tests are presented in Table 4 and Fig. 5. The Young's modulus for all samples ranged between 3.27 and 8.5 GPa. On average, femoral bone has a high yield strain of 1.6% and failure strain of 7.5% (Fig. 5, Table 4). The MANCOVA showed a significant overall effect of femur length on the biomechanical properties of the femur (Wilks' lambda=0.19; *F*_7,13_=7.71; *P*=0.001). Whereas population differences were significant (Wilks' lambda=0.39; *F*_7,13_=2.91; *P*=0.046), differences between sexes were not (Wilks' lambda=0.53; *F*_7,13_=1.68; *P*=0.20). The interaction between population and sex was also non-significant (Wilks' lambda=0.85; *F*_7,13_=0.34; *P*=0.92). Subsequent ANCOVAs showed that populations differed significantly in *I*_min_ (*F*_1,19_=17.65; *P*<0.001) and diameter (*F*_1,19_=20.58; *P*<0.001). Inspection of the marginal means showed that individuals from the core had a higher minimal principal axis value (i.e. lowest distribution of mass) and diameter compared with those of the edge.

**Table 4.**
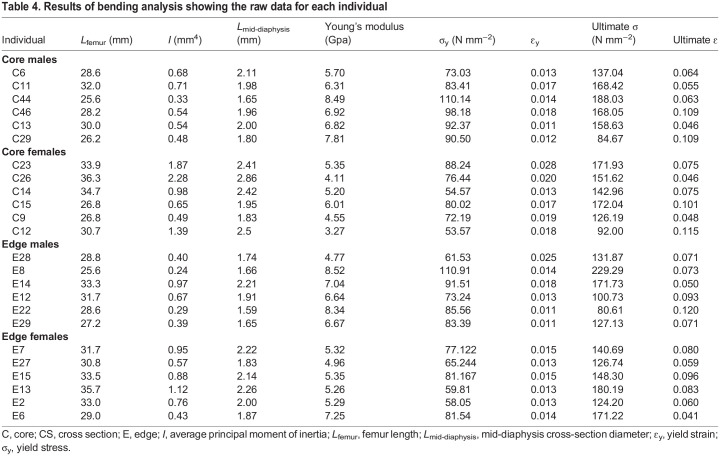
Results of bending analysis showing the raw data for each individual

## DISCUSSION

The growth of the long bones in frogs differs from that described for mammals and birds in that periosteal ossification dominates, with endochondral ossification observed only on the edges of erosion bays at the epiphyses (Fox and Irving, 1950; Dickson, 1982; Felisbino and Carvalho, 2002; Rozenblut and Ogielska, 2005; Haines, 2008). Cortical thickness changes, on the other hand, are due to the periosteal activity around the diaphysis (Dickson, 1982; Dell'Orbo et al., 1992; Felisbino and Carvalho, 1999, 2001; Rozenblut and Ogielska, 2005; Erismis and Chinsamy, 2010). Dell'Orbo et al. (1992) stated that bone diameter increased with age without a significant increase in the diameter of the medullary cavity, and concluded that thickening of the diaphyseal cortex was the principal driver of the widening of the diaphysis. We did observe that, independent of variation in size, individuals from the core of the range had a greater cortical thickness compared with edge individuals.

Endochondral ossification has been observed to occur late in the development of aquatic and semi-aquatic frogs and is restricted to the boundary zones of metaphyseal cartilage (Rozenblut and Ogielska, 2005; Miura et al., 2008). This late ossification and trabecular bone formation in the epiphyses occur in conjunction with the cessation of bone elongation (Kemp and Hoyt, 1969; Felisbino and Carvalho, 2001). This is the likely explanation for the absence of trabecular bone in younger individuals in our study. Dell'Orbo et al. (1992) also reported the absence of trabecular bone in *Pelophylax kl. esculentus*. In contrast, some trabecular bone was observed in *Peplophylax caralitanus* from the age of 6 years onwards (Erismis and Chinsamy, 2010) with well-developed struts being present in older individuals. Felisbino and Carvalho (2001) only observed bone trabeculae resulting from endochondral ossification in *Lithobates catesbeianus* that were already almost fully grown, similar to the observations of Rozenblut and Ogielska (2005) in *P. kl. esculentus*. Felisbino and Carvalho (2001) hypothesized that trabecular formation only occurs with an increased body size and weight in adults, and functions to reinforce the ends of the bones. In accordance, in *X. laevis* we found that the amount of trabecular bone increased with age (i.e. femur length). Moreover, trabecular bone first appeared in the condyle (distal epiphysis) which may reflect the higher mechanical demands on this structure. The earlier onset of the deposition of calcium phosphate in the articular cartilage of the knee joint (distal femur and proximal tibiofibular) observed by Felisbino and Carvallho (2001, 2002) in *Lithobates catesbeianus* also suggests a possible high mechanical stress in this region. Trabecular bone structure was, however, not different between sexes nor between populations, suggesting that it is of no advantage to individuals at the edge of range. This may be because trabecular bone develops too late in the ontogeny to be a significant factor in the response of the bone to higher or more frequent loading. Morphologically and functionally, the epiphyses of the long bones in frogs are different to those of mammals and birds (e.g. Ruimerman et al., 2005; Pontzer et al., 2006; Barak et al., 2008, 2011; Kivell, 2016), where the formation of trabecular bone enhances the mechanical properties of the bone. Moreover, a consistent relationship between trabecular bone architecture with mechanical properties is not necessarily true and depends on animal location, bone function and mode of loading (e.g. Barak et al., 2010).

### Long bone architecture, material, and mechanical properties

The cross-section of the femora of *X. laevis* does not have a visible crest along the length of the diaphysis similar to what has been reported for other aquatic frogs (Vera et al., 2020). However, aquatic and semi-aquatic frogs were predicted by the same authors to have a round diaphysis. The mean eccentricity (0.5) we observed in our CT scans shows that the diaphysis of *X. laevis* is not round but elliptical, with a laterally elongated shape (Fig. 1). It has been hypothesized that a circular geometry is an adaptation to torsion (Broshko, 2014) or multidirectional bending (Demes and Carlson, 2009). A more elliptical cross-section, in contrast, is better suited to resist bending in one direction. This geometry appears to be conserved as there were no differences between sexes or populations. However, significant differences between core and edge individuals were detected for most of the other cortical parameters measured. Core and edge individuals differ in muscle mass and muscle cross-sectional area, with edge individuals showing more strongly developed muscles (e.g. femur retractor, knee extensor, ankle extensor and flexor; Padilla et al., 2019). Our results show, however, that the cortical bone is thinner, and that edge individuals have lower cortical bone parameters and a lower bone mineral density, suggesting that their bones are less resistant to bending. Although this pattern could be indicative of fast locomotion, we have no field data on locomotor speed that could test this idea.

In addition to differences between populations, we also found significant differences between sexes. Our analysis showed that females have a greater total cross-sectional and cortical bone area, as well as greater mean second polar moments of area for a given femur length. In previous studies, sex-specific differences in organ size and muscle architecture have been highlighted (Padilla et al., 2019). Interestingly, females had heavier muscles but not a greater physiological muscle cross-sectional area compared with males for a given femur length. Females further invest more energy in reproductive output compared with males (Shine, 1979; Courant et al., 2017) and are larger and more corpulent. The greater corpulence of females (i.e. higher weight for a given size) may explain the greater investment in cortical bone, allowing females to resist these greater external forces (body weight) during terrestrial locomotion. This is in accordance with the fact that muscle cross-sectional area does not differ between males and females, suggesting that the cortical structure of the femur in *X. laevis* responds mostly to differences in the external (gravitational) forces experienced during terrestrial locomotion.

Bone mineral density has a tight relationship with mechanical properties (e.g. Currey, 1990, 1999). Currey (1999), for example has shown a strong relationship of calcium content (proxy for mineral content and comparable in our study with bone density) and Young's modulus (bending strength) in a large sampling of different bone types and species. *Xenopus laevis* females did show a lower BMD than males which is reflected in their lower Young's modulus. Edge individuals also had a significantly lower BMD compared with core individuals. BMD has been shown to improve bone strength (e.g. Turner, 2006; Currey, 2002; Ammann and Rizzoli, 2003). In frogs, jumping is mechanically more challenging than swimming (e.g. Lutz and Rome, 1994; Calow and Alexander, 1973). Indeed, jumping has been hypothesized to subject frog hind limb bones to high torques requiring a high bone bending strength (e.g. Blob and Biewener, 1999; Blob et al., 2014). Blob and co-authors (2014) showed that *in-vitro* strains measured on the femora of frogs were indeed indicative of bending (e.g. Biewener et al., 1983; Blob and Biewener, 1999). Moreover, Wilson et al. (2009) showed that anuran hind limb bones generally show high yield stresses in bending. Our three-point bending experiment showed that the Young's modulus of the femora in *X. laevis* ranges from 3.2 to 8.5 GPa. These values fall within the range observed for fish bone (5–8 GPa; Atkins et al., 2014), yet are somewhat lower than values documented for mammalian bone (5–25 GPa; Currey, 1999, 2002; Shahar et al., 2007). The bending yield stress in *X. laevis* ranged from 55 to 110 MPa and is somewhat lower than values observed and reported for femora of other vertebrates (birds, mammals, reptiles) (96–316 MPa; Currey, 1987, 2002; Erickson et al., 2002). The yield strain and stress values recorded here are also somewhat lower than previous data obtained for frogs (Wilson et al., 2009). Differences between the results obtained here and those of previous studies may be due to a variety of factors, including differences in the loading rate (lower in our study), the span width used in the three-point bending test, preservation artifacts, the specific set-up used and phylogenetic or ecological differences between species. Future studies would benefit from estimates of *in vivo* loading rates, adapting the span to the size of the bone, and the use of fresh bone to better understand if the values reported here are good estimates of *in vivo* whole-bone mechanical properties. Only then will we be able to fully understand their functional and ecological relevance.

### Conclusions

Our study showed important differences in the cortical bone geometry between frogs from the core and the edge of the range of an expanding invasive population of *X. laevis*. Moreover, cortical bone structure and bone biomechanics differed significantly between sexes. However, our hypothesis that all range edge frogs, as well as males, would show adaptations to resist higher and more frequent loading was not supported. In contrast, individuals from the core showed a higher bending resistance compared with individuals from the range edge, which is possibly due to their relatively higher body mass. As many differences have been observed between core and edge individuals in expanding populations, including for *X. laevis*, the fact that edge individuals of this species are able to disperse further and more frequently while they show a lower bending resistance might be explained by a more important behavioral tendency to move in individuals from the range edge.

## Supplementary Material

10.1242/jexbio.246419_sup1 Supplementary information
