# Supplementary material for: Femoral bone structure and mechanics at the edge and core of an expanding population of the invasive frog Xenopus laevis
Source: J Exp Biol. 2024 Jul 11;227(13):jeb246419. doi: 10.1242/jeb.246419 (PMC11418183; doi:10.1242/jeb.246419)
Supplement: Supplementary information [file jexbio-227-246419-s1.pdf]

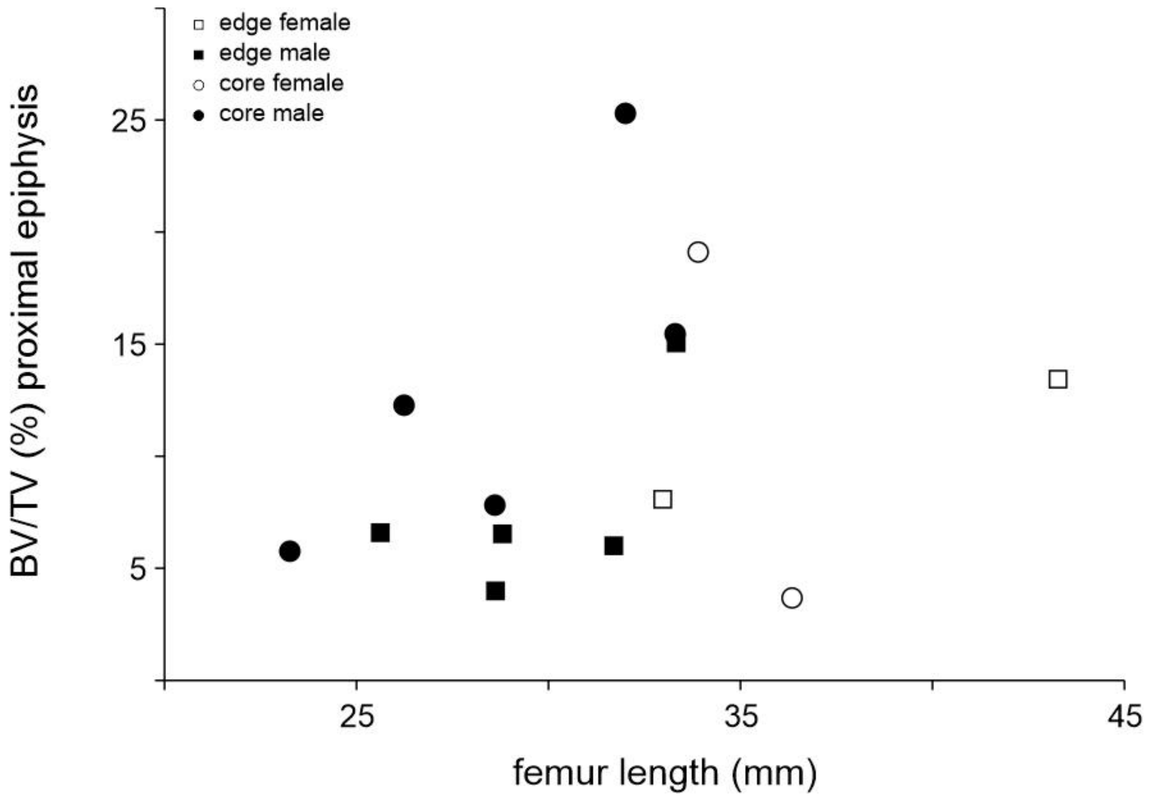

Fig. S1. Bone volume relative to total tissue volume in the proximal epiphysis of *Xenopus laevis* in function of femur length. Black symbols represent males, white symbols represent females. Squares represent edge individuals, circles represent core individuals.

Journal of Experimental Biology • Supplementary information

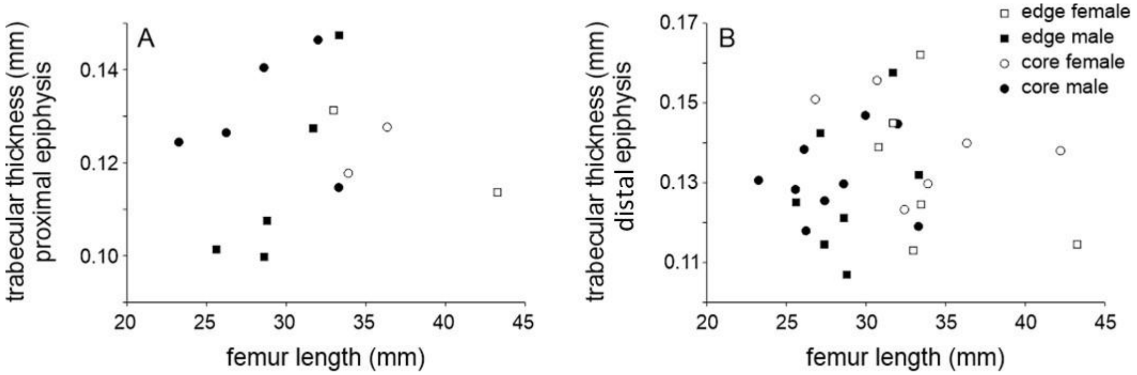

Fig. S2. Trabecular thickness in the proximal (A) and distal (B) epiphyses of *Xenopus laevis* in function of femur length. Black symbols represent males, white symbols represent females. Squares represent edge individuals, circles represent core individuals.

Journal of Experimental Biology • Supplementary information

Table S1. Table summarizing the presence/absence of trabecular bone in the epiphyses in *Xenopus laevis* femora. Individuals are ranked according to their length. Grey colored cells highlight individuals with no or few trabeculae; black colored cells highlight individuals with many trabeculae. Only individuals indicated in black were used in the quantitative analysis.

**Core males**																		
Individual	C20	C41	C44	C27	C29			C46	C6		C13	C11		C38				
Femur length (mm)	23.2	25.2	25.5	26.1	26.2			28.1	28.6		29.9	32.0		33.3				
Anterior epiphysis																		
Posterior epiphysis																		
**Core females**																		
Individual					C15	C9					C12	C24			C23	C14	C26	C8
Femur length (mm)					26.8	26.8					30.7	32.4			33.9	34.7	36.3	42.2
Anterior epiphysis																		
Posterior epiphysis																		
**Edge males**																		
Individual	E25	E10	E8				E17	E29	E22	E28		E12		E14				
Femur length (mm)	24.6	25.3	25.6				27.3	27.6	28.6	28.8		31.7		33.3				
Anterior epiphysis																		
Posterior epiphysis																		
**Edge females**																		
Individual									E6		E27	E7	E2	E11	E15	E1	E13	E16
Femur length (mm)									28.5		30.8	31.7	32.9	33.4	33.4	34.9	35.6	43.2
Anterior epiphysis																		
Posterior epiphysis																		

C = Core; E = Edge

Journal of Experimental Biology • Supplementary information

Table S2. Tests of normality for the Log_{10}-transformed variables.

	Shapiro Wilk	d.f.	*P*
Femur length (mm)	0.948	11	0.61
Mean total cross-sectional tissue area (mm^2)	0.964	11	0.82
Mean total cross-sectional bone area (mm^2)	0.916	11	0.29
Cross-sectional thickness (mm)	0.896	11	0.17
Mean polar moment of inertia (mm^4)	0.953	11	0.69
Average maximal principal moment of inertia (mm^4)	0.956	11	0.72
Average minimal principal moment of inertia (mm^4)	0.952	11	0.67
Eccentricity	0.953	11	0.68
Tissue volume - distal (mm^3)	0.938	11	0.49
Bone volume - distal (mm^3)	0.991	11	1.00
Bone volume/tissue volume - distal (%)	0.981	11	0.97
Trabecular thickness - distal (mm)	0.990	11	1.00
Trabeculae number - distal (mm^{-1})	0.967	11	0.85
Trabecular separation - distal (mm)	0.986	11	1.00
Bone mineral density (g/cm^3)	0.896	11	0.17
Average moment of inertia (mm^4)	0.954	11	0.70
Cross sectional mid-diaphysis diameter (mm)	0.958	11	0.74
Young's modulus (GPa)	0.934	11	0.45
Yield stress σ (N/mm^2)	0.907	11	0.23
Yield strain **ε**	0.861	11	0.06
Ultimate stress (N/mm2)	0.954	11	0.69
Ultimate strain	0.958	11	0.74

Journal of Experimental Biology • Supplementary information

Table S3. Results of the Levene's tests for homogeneity of variance

	F	d.f.	*P*
Mean total cross-sectional tissue area (mm^2)	1.918	3,32	0.147
Mean total cross-sectional bone area (mm^2)	0.682	3,32	0.570
Cross-sectional thickness (mm)	0.890	3,32	0.457
Mean polar moment of inertia (mm^4)	2.038	3,32	0.128
Average maximal principal moment of inertia (mm^4)	1.999	3,32	0.134
Average minimal principal moment of inertia (mm^4)	2.375	3,32	0.088
Eccentricity	1.286	3,32	0.296
Bone mineral density (g/cm^3)	0.074	3,32	0.973
Tissue volume - distal (mm^3)	3.200	3,7	0.093
Bone volume - distal (mm^3)	1.381	3,7	0.325
Bone volume/tissue volume - distal (%)	5.344	3,7	0.031
Trabecular thickness - distal (mm)	3.751	3,7	0.068
Trabeculae number - distal (mm^{-1})	2.400	3,7	0.153
Trabecular separation - distal (mm)	2.006	3,7	0.202
Average moment of inertia (mm^4)	1.228	3,7	0.369
Cross sectional mid-diaphysis diameter (mm)	0.598	3,7	0.636
Young's modulus (GPa)	0.852	3,20	0.482
Yield stress σ (N/mm^2)	3.242	3,20	0.044
Yield strain **ε**	1.319	3,20	0.296
Ultimate stress (N/mm2)	0.852	3,20	0.482
Ultimate strain	0.196	3,20	0.898

Note that yield stress does not show homogeneity of variance.

Journal of Experimental Biology • Supplementary information

Table S4. Trabecular variables for the anterior and posterior epiphyses of *Xenopus laevis* femora.

Ind	**Tissue volume** (mm^3)	**Bone volume** (mm^3)	**BV/TV** (%)	**Trabecular thickness** (mm)	**Trabeculae number** (mm^{-1})	**Trabecular separation** (mm)
Proximal epiphysis						
**C20**	4.91	0.28	5.76	0.12	0.46	0.86
**C6**	3.48	0.27	7.82	0.14	0.56	0.64
**C11**	10.11	2.56	25.29	0.15	1.73	0.56
**C29**	5.21	0.64	12.27	0.13	0.97	0.58
**C38**	6.84	1.06	15.46	0.12	1.35	0.71
**C23**	11.19	2.14	19.10	0.12	1.62	0.38
**C26**	19.2	0.72	3.67	0.13	0.29	2.16
**E16**	15.82	2.13	13.44	0.11	1.18	0.47
**E2**	6.61	0.53	8.08	0.13	0.62	1.10
**E8**	2.35	0.16	6.58	0.10	0.65	0.71
**E12**	2.68	0.16	6.01	0.13	0.47	0.51
**E14**	10.49	1.58	15.06	0.15	1.02	0.82
**E22**	1.70	0.07	3.99	0.10	0.40	0.46
**E28**	3.31	0.22	6.53	0.11	0.61	0.60
Distal epiphysis						
**C20**	6.41	0.36	5.64	0.13	0.43	1.10
**C6**	14.44	2.08	14.37	0.13	1.11	0.67
**C11**	21.19	5.63	26.58	0.15	1.84	0.65
**C13**	10.59	1.22	11.47	0.15	0.78	0.99
**C27**	17.78	1.91	10.73	0.14	0.78	1.27
**C29**	15.52	1.62	10.41	0.12	0.88	0.82
**C38**	23.29	2.77	11.89	0.12	1.00	0.79
**C41**	7.61	0.46	6.09	0.13	0.49	0.90
**C44**	8.49	0.84	9.93	0.13	0.77	0.86
**C8**	41.26	7.67	18.60	0.14	1.35	0.68
**C23**	49.78	9.34	18.75	0.13	1.45	0.67
**C9**	4.90	0.69	14.16	0.15	0.94	0.58
**C12**	14.43	1.67	11.59	0.16	0.75	0.86
**C24**	29.54	1.85	6.27	0.12	0.51	1.34
**C26**	54.63	4.22	7.72	0.14	0.55	1.26
**P15**	14.03	1.43	10.22	0.13	0.82	1.09
**E16**	43.34	5.93	13.69	0.12	1.20	0.57
**E2**	16.91	3.22	19.06	0.11	1.69	0.51
**E7**	5.30	0.42	7.95	0.15	0.55	0.55
**E11**	13.64	0.72	5.28	0.16	0.33	0.92
**E27**	13.32	1.15	8.62	0.14	0.62	1.13
**E8**	17.61	1.79	10.15	0.13	0.81	0.80

Journal of Experimental Biology • Supplementary information

**E12**	10.26	1.10	10.73	0.16	0.68	0.96
**E14**	18.16	2.50	13.77	0.13	1.04	0.75
**E17**	43.34	5.93	13.69	0.12	1.20	0.57
**E22**	5.19	0.65	12.47	0.12	1.03	0.56
**E28**	8.41	1.33	15.84	0.11	1.48	0.43
**E29**	5.90	0.54	9.15	0.14	0.64	0.70

BV/TV = bone volume divided by tissue volume.

Journal of Experimental Biology • Supplementary information
